# Age-Specific Etiology of Severe Acute Respiratory Infections and Influenza Vaccine Effectivity in Prevention of Hospitalization in Russia, 2018–2019 Season

**DOI:** 10.1007/s44197-021-00009-1

**Published:** 2021-10-28

**Authors:** Anna Sominina, Daria Danilenko, Andrey Komissarov, Maria Pisareva, Tamila Musaeva, Mikhail Bakaev, Olga Afanasieva, Kirill Stolyarov, Elizaveta Smorodintseva, Elena Rozhkova, Elena Obraztsova, Elena Dondurey, Dmitry Guzhov, Veronica Timonina, Ekaterina Golovacheva, Olga Kurskaya, Alexander Shestopalov, Svetlana Smirnova, Alexander Alimov, Dmitry Lioznov

**Affiliations:** 1 Smorodintsev Research Institute of Influenza, Prof. Popova Str., 15/17, 197376 Saint Petersburg, Russia; 2 Federal Research Center for Fundamental and Translational Medicine, Timakova, 2 Str., 630117 Novosibirsk, Russia; 3 Yekaterinburg Research Institute of Viral Infections of FBRI SSC VB “Vector”, Letnyaya 23 Str., Sverdlovsk Region, 620030 Yekaterinburg, Russia; 4 Children’s City Hospital Named After St. Olga, Zemledelcheskaya Str., 2, 194156 St. Petersburg, Russia

**Keywords:** Influenza, Respiratory viruses, Hospitalization, Vaccine effectiveness

## Abstract

The expansion and standardization of clinical trials, as well as the use of sensitive and specific molecular diagnostics methods, provide new information on the age-specific roles of influenza and other respiratory viruses in development of severe acute respiratory infections (SARI). Here, we present the results of the multicenter hospital-based study aimed to detect age-specific impact of influenza and other respiratory viruses (ORV). The 2018–2019 influenza season in Russia was characterized by co-circulation of influenza A(H1N1)pdm09 and A(H3N2) virus subtypes which were detected among hospitalized patients with SARI in 19.3% and 16.4%, respectively. RSV dominated among ORV (15.1% of total cases and 26.8% in infants aged ≤ 2 years). The most significant SARI agents in intensive care units were RSV and influenza A(H1N1)pdm09 virus, (37.3% and 25.4%, respectively, of PCR-positive cases). Hyperthermia was the most frequently registered symptom for influenza cases. In contrast, hypoxia, decreased blood O_2_ concentration, and dyspnea were registered more often in RSV, rhinovirus, and metapneumovirus infection in young children. Influenza vaccine effectiveness (IVE) against hospitalization of patients with PCR-confirmed influenza was evaluated using test-negative case–control design. IVE for children and adults was estimated to be 57.0% and 62.0%, respectively. Subtype specific IVE was higher against influenza A(H1N1)pdm09, compared to influenza A(H3N2) (60.3% and 45.8%, respectively). This correlates with delayed antigenic drift of the influenza A(H1N1)pdm09 virus and genetic heterogeneity of the influenza A(H3N2) population. These studies demonstrate the need to improve seasonal influenza prevention and control in all countries as states by the WHO Global Influenza Strategy for 2019–2030 initiative.

## Introduction

Building on its 70 years of global health leadership, the WHO developed the Global Influenza Strategy for 2019–2030 to enhance global and national pandemic preparedness, to combat the ongoing threat of zoonotic influenza, and to improve seasonal influenza prevention and control in all countries [1]. Despite the progress made, many challenges and gaps remain. Recent studies have shown that respiratory viral infections, including those due to the influenza virus, increase the risk of heart attack and stroke in the 3 days following infection [2], and frequently exacerbate chronic obstructive pulmonary disease [3]. The link between severe pneumonia and influenza has also been further investigated. Other studies have shown that up to 50% of pneumonia cases are linked to respiratory viruses, and up to 45% of pneumonia cases in children show evidence of viral–bacterial co-infection [1].

The newly established Global Influenza Hospital Surveillance Net (GIHSN) has opened additional opportunities for strengthening of the clinical and laboratory surveillance for influenza and other respiratory viruses. The system provides opportunities for determination of age-related features of severe acute respiratory infection (SARI) etiology, including: illness severity, with identification of dominating agents; comorbidities leading to hospitalization; and assessment of risk factors on the clinical and virological levels. The GIHSN system enables an objective assessment of the effectiveness of influenza vaccines and antivirals in preventing hospitalization [4–6]. Moreover, subtle analysis of the antigenic and genetic structure of influenza viruses makes it possible to determine whether the viruses circulating in a specific season or country match the strains introduced into the vaccine composition.

An important step in promoting these studies is the introduction of Next Generation Sequencing (NGS) technology, which permits whole genome virus sequencing to search for viral determinants responsible for pathogenicity [7]. There is growing interest in detailed genomic analysis, at the quasispecies level, of viruses causing SARI [8]. Recently, it was found that quasispecies have an important role in the pathophysiology of influenza infection and are factors affecting virulence and virus spread [9, 10].

The objectives of the study were: determination of age-specific impact of influenza and other respiratory viruses (ORV), leading for admission with SARI; and evaluation of influenza vaccine effectiveness in prevention of hospitalization during the whole period of increased influenza activity (from the week 52.2018 to the week 20.2019) in three megalopolises of Russia.

## Materials and Methods

### Principles and Organizational Structure of the Investigation

The principles and algorithm of the study were fully consistent with those adopted by the Global Influenza Hospital Surveillance Network (GIHSN) [11]. Clinical and laboratory (rRT-PCR) monitoring of influenza and other respiratory infections (ORI), among hospitalized patients, was conducted at nine participating hospitals in three large, geographically separated Russian cities located in the Northwestern, Siberian, and Ural Districts (St. Petersburg, Novosibirsk and Ekaterinburg, respectively). Novosibirsk and Ekaterinburg were chosen as highly populated, geographically remote large cities located in different climatic zones, acting as major transport hubs and being cultural and educational centers in the Urals and Siberia. The Smorodintsev Research Institute of Influenza (St. Petersburg) was the Coordination Centre of the study where all the data accumulated in electronic database.

All patients with SARI, who met the criteria for inclusion in the study, were examined by clinicians participating in the study. Personal electronic standardized Core questionnaire for children less than 5 years of age and for patients 5 years or more, were completed by physicians and submitted to the Electronic Database of the Smorodintsev Research Institute of Influenza after specific control. All patients meeting the inclusion criteria were swabbed for PCR detection of influenza and other respiratory viruses (ORV), including RSV, rhinovirus, parainfluenza, adenovirus, metapneumovirus, coronavirus, and bocavirus.

### The Time and Place of the Study

The study period covered the whole period of influenza activity and lasted about 5 months starting from week 52.2018, when the first influenza cases were detected by real-time RT-PCR (PCR) in hospitals under surveillance. The study was completed on week 20.2019, when no further SARI patients with laboratory-confirmed influenza (LCI) were detected in any wards of hospitals included in the study.

### Case Definition

The European CDC's influenza case definitions were used here for patient selection [12] with minor changes. Severe acute respiratory infection (SARI) cases are defined as patients with an acute respiratory infection who have one or more respiratory symptoms: cough, sore throat, shortness of breath, stuffy nose, onset within the last 7 days and require hospitalization. They included also a combination of systemic symptoms such as a history of fever, or measured fever of ≥ 38 °C, malaise, headache and myalgia. Additionally, number of severity criteria of disease was used in analysis of the study results including: hyperthermia (>39 °C), hypoxia (SpO_2_ less than 94%), lethargy, O_2_ in the blood less than normal (95–97%), blood urea more than normal (1.8–6.4 mmol/l for children < 14 years, 2.5–6.4 mmol/l for the patients aged ≥ 14 years), hemorrhagic syndrome, ICU admission, mechanical ventilation.

### Criteria for Study Inclusion

All patients with influenza like illnesses (ILI) symptoms requiring hospitalization were classified as SARI. Other inclusion requirements were: disease duration not more than 7 days from the onset of (ILI) symptoms; and hospitalization for the previous 24–48 h. Nasal and throat samples were obtained from all children younger than 14 years old. Swabs, from the nasopharynx and oropharynx, were taken (within the first 48 h of patient admission) from each patient aged ≥ 14 years.

### Exclusion Criteria

Cases were excluded from the study if the patient has not agreed to participate in the study, if the symptoms of the disease did not match the signs of ILI, if the onset of ILI was more than for 7 days, if he exposed to influenza in an institutional setting, or if the patient resided outside cities included in the study.

### Influenza Virus Isolation, Identification, and Antigenic Analysis

Were performed according to the WHO Manual [13] and appropriate guidelines approved in Russia [14]. Influenza viruses isolated during the epidemic period 2018–2019 were characterized antigenically in hemagglutination inhibition test (HI) or in microneutralization assay (MN) with the strain specific post infectious ferret antisera kindly provided by Dr. John McCauley (WHO CC at the Francis Crick Institute in London).

### PCR Detection of Influenza and Other Respiratory Viruses

RNA was isolated from clinical samples using the AmpliSense^®^ RIBO-prep kit (InterLabService, Russia) or the RNeasy Mini kit (QIAGEN). Reverse transcription of RNA was performed using the Reverta-L kit (InterLabService, Russia) or the OneStep RT-PCR Kit (QIAGEN) with CDC primers and probes. PCR for influenza A and B viruses was performed using the AmpliSense^®^ Influenza virus A/B-FL kit (InterLabService, Russia). Detection of other respiratory viruses was performed using the multiplex AmpliSense^®^ ARVI-screen-FL kit (InterLabService, Russia). All real-time PCR reactions were carried out using a Rotor-Gene 6000 (Corbett Research, Australia) or a CFX96 Touch™ Real-Time PCR Detection System (BIO-RAD, USA).

### Effectiveness of Influenza Vaccine (IVE)

For the 2018–2019 epidemic season, in preventing patient hospitalization, was evaluated using test-negative case-control design [15].

PCR-confirmed influenza A(H1N1)pdm09, A(H3N2) or B virus infection registered in the indicated period were defined as cases. Influenza negative cases according to PCR results were defined as control. IVE was calculated as IVE = (1 − OR) × 100.

Russian trivalent influenza polymeric subunit vaccine “Sovigripp”, containing 5 µg of HA per dose each of influenza A(H1N1)pdm09 and A(H3N2) virus, 11 µg of HA per dose of influenza B virus and 500 µg of the adjuvant “Sovidon” was used mainly for the immunization of the population in St. Petersburg, Ekaterinburg, and Novosibirsk in 2018–2019 season. The vaccine “Sovigripp” for children over 3 years old, adolescents and adults (without age limitation) injected once intramuscularly in dose of 0.5 ml. This vaccine was included in the National Calendar of Vaccination and was purchased from the Federal Budget. Other domestic and foreign influenza vaccines were used rarely and collectively comprised less than 1% of patients being vaccinated in this study. All vaccines were trivalent and included the strains recommended by WHO experts for Northern hemisphere for this season: A/Michigan/45/15(H1N1)pdm09, A/Singapore/INFIMH-16-0019/16(H3N2) and B/Colorado/06/17 (Victoria lineage).

All IVE estimates were stratified by influenza activity period (from week 52.2018 to week 20.2019). Cases with laboratory-confirmed influenza A(H1N1)pdm09, A(H3N2) or B virus in the indicated period were included as positive cases. Cases were excluded from analysis if they were ineligible for influenza immunization (children younger than 3 years), reported as epidemiologically linked (but not laboratory-confirmed), exposed to influenza in an institutional setting, or if they resided outside cities included in the study. IVE was calculated using data from cases with a recorded (yes/no) immunization status only; cases with an unknown/missing status were excluded. IVE estimates were adjusted for age (3–6, 7–14, 15–17 and ≥ 18 years.

### Statistical Analysis

Statistical analyses were performed using Statistica 10.0 nonparametric criteria, bivariate method chi-square (*p* values less than 0.05 were considered to be statistically significant). For odds ratio, R software version 3.7 was used. Associations between categorial variables were estimated with Fisher test. All confidence intervals were 95%. An association was considered statistically significant if *p* < 0.05.

### Ethical Aspects of the Study

Investigation performed in accordance with the principles of Good Clinical Practice (GCP). The Local Ethics Committees approved the study before its initiation. Patient consent to study involvement was a research prerequisite.

## Results

### Patient Recruitment and Distribution, by Age Group

After obtaining informed consent and initial clinical selection, a total of 3057 of 3084 hospitalized patients were included in the study, among them 2131 (69.7%) children aged ≤ 17 years (years), and 926 (30.3%) adults. The hospitalization rate for children aged ≤ 2 years was the highest (39.8%), compared to other groups: 3–6 years (18.9%); 7–14 years (8.6%); 15–17 years (2.4%); 18–64 years (26.9%); and ≥ 65 years (3.4%). The overall number of patients included in the study comprised 9.2% of the total number of the patients hospitalized with SARI in all hospitals of St.Petersburg, Ekaterinburg, and Novosibirsk.

### Profile of Patients Included in the Study

Positive PCR results for influenza were obtained in 1108 (36.2%) cases from 3057 patients included in the study; ORV were detected in 1169 (38.2%) patients, and 973 (31.8%) patients were negative for the respiratory viruses tested. A total of 709 (64%) cases of laboratory-confirmed influenza (LCI) were registered among children, and 399 (36%) cases were in adults.

### Burden of Influenza and ORV

Influenza A(H1N1)pdm09 and A(H3N2) were detected in 19.3% and 16.4% of cases, respectively. This season, influenza B/Yamagata and B/Victoria lineages were very rare: 0.3% and 0.1% of cases, respectively. Influenza A(H3N2) was more frequent in adult patients, whereas influenza A(H1N1)pdm09 predominated in children ≤ 14 years old (Table 1). ORV affected mostly young children (45.8–58.9% of cases, compared to 12.0–16.5% in adults), with RSV being the most significant pathogen. The hospitalization rate with ORV compared to influenza was significantly higher in children compared to adults (*p* = 0.00001). RSV comprised 15.1% of all investigated patients and 26.8% of patients aged 0–2 years. This age group was the most affected by RSV, parainfluenza, metapneumovirus and adenovirus with significant differences (*p* < 0.05) from age group 7–14 years old and more. Rhinovirus was the second most frequently detected ORV, causing 8.2% of hospitalized cases in total. Metapneumovirus was detected mostly in young children aged 0–2 or 3–6 years. Other respiratory viruses were less often causative agents of SARI.

**Table 1:** Age dependent rate of influenza and other respiratory viruses detection in hospitalized patients, season 2018–2019

Age group	Number of cases			Percent of positive for viruses cases^a^														
	Included in the study	Not identified cases	Influenza and ARI viruses detected	H1N1 pdm09	H3N2	A^a^	BYam	BVic	B^a^	Total influenza	PIV	AdV	RSV	MpV	CoV	BoV	RhV	TotalORI
0–2	1218	281	937	17.9	9.9	0.0	0.2	0.1	0.0	28.0	3.9	3.2	26.8	7.4	3.9	3.5	10.1	58.9
3–6	577	128	449	24.4	15.6	0.2	0.3	0.0	0.2	40.7	2.3	2.1	16.5	8.5	2.9	2.3	11.3	45.8
7–14	262	102	160	23.3	16.0	0.4	0.8	0.0	0.0	40.5	1.5	0.8	5.3	3.4	2.7	1.1	8.8	23.7
15–17	74	38	36	14.9	21.6	0.0	0.0	0.0	0.0	36.5	1.4	2.7	2.7	1.4	2.7	0.0	2.7	13.5
18–64	823	384	439	18.0	23.8	0.2	0.2	0.2	0.1	42.6	1.7	1.0	2.2	1.5	1.5	0.0	4.3	12.0
≥ 65	103	40	63	10.7	35.0	0.0	1.0	0.0	0.0	46.6	2.9	0.0	4.9	1.9	3.9	0.0	2.9	16.5
Total	3057	973	2084	19.3	16.4	0.1	0.3	0.1	0.1	36.2	2.7	2.1	15.1	5.3	2.9	1.9	8.2	38.2

In this and following tables percent of positive cases was calculated as the ratio of the number of identified cases to the number of included in the study and swabbed patients by age groups

^a^Subtype/lineage is not determined

### Etiology of SARI Among Intensive Care Unit (ICU) Patients

Overall, 89 (2.9%) patients within the study were placed in the ICU, including 48 (54%) children aged 0–2 years, 19 (21.3%) children aged 3–6 years, and 10 (11.2%) patients aged 7–14 years. The number of patients in other age groups was small (2–5 patients). Convulsions, hyperthermia, cardiac failure, and respiratory failure were the top reasons for ICU admission. A total of 59 patients were positive for different respiratory viruses: 21 (35.6%) patients were positive for influenza viruses; and 45 (76.3%) were positive for ORV. Seven (11.9%) patients had mixed infection (mostly influenza and RSV). RSV and influenza A(H1N1)pdm09 appeared to be the main etiological agents among ICU admissions (37.3% and 25.4%, respectively).

**Fig. 1:**
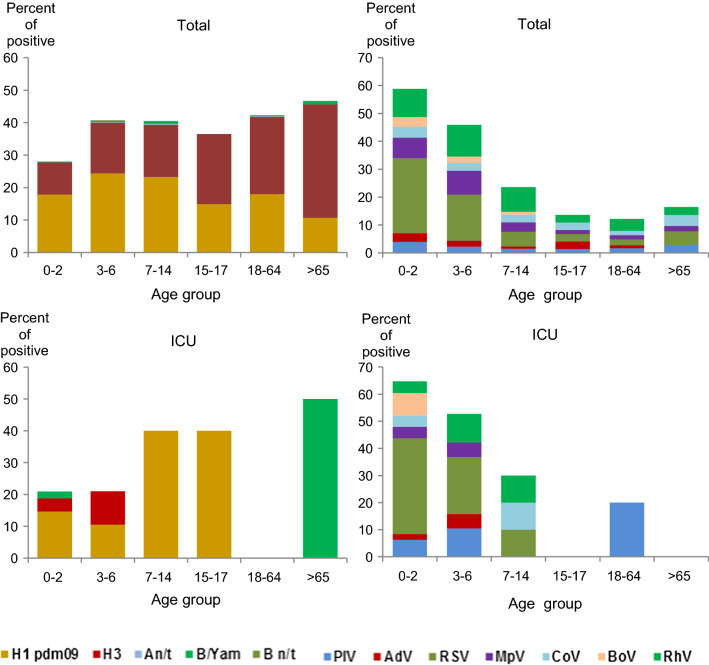
Age dependent rate of influenza (left) and ORV (right) detection among total number of patients and those admitted to ICU. Age group (number of patients total/ICU): 0–2 years (1218/48), 3–6 years (577/19), 7–14 years (262/10), 15–17 years (74/5), 18–64 years (823/5), ≥ 65 years (103/2)

Analysis of age-related etiology of SARI in the ICU showed that the percent of influenza cases increased with age from 21% in young patients (0–2 years) to 40% in school children. In contrast, the ORV impact decreased from 64% to 20%. The most affected group was young children (Fig. 1).

### Monitoring of Influenza and ORV Activity in the 2018–2019 Season

**Fig. 2:**
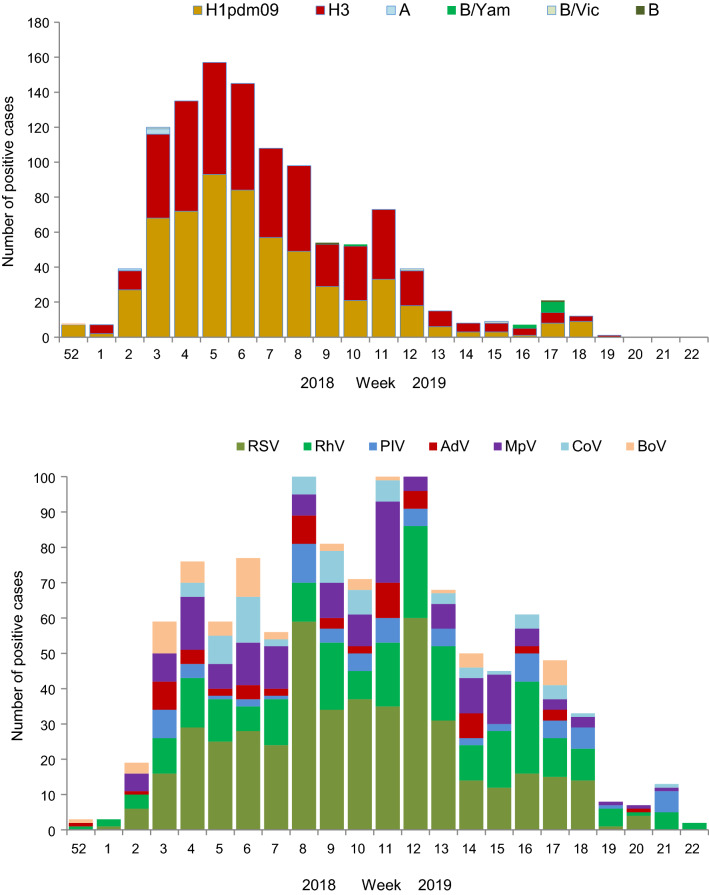
Weekly monitoring of influenza, RSV, rhinoviruses, parainfluenza, adenoviruses, metapneumoviruses, coronaviruses and bocaviruses among patients admitted in hospitals included in the study

The first influenza A cases were identified among hospitalized patients on week 52.2018. During the following weeks, the number of influenza cases increased progressively until peaking on week 5.2019. A decrease in influenza activity was gradual and followed until week 20.2019, when no influenza cases were detected in the hospitals. Co-circulation of influenza A(H1N1)pdm09 and A(H3N2) viruses, with a single influenza B virus detection, was observed during the entire season. In addition, RSV dominated practically during the whole period of increased influenza activity (from week 2 to week 18). As in the previous seasons, rhinoviruses and metapneumoviruses were next in importance as causative agents among hospitalizations. Parainfluenza viruses, adenoviruses, coronaviruses, and bocaviruses were detected less often (Fig. 2).

### Influenza and ORV in Females and Males

In total, 1552 (50.8%) males and 1505 (49.2%) females were included in the study, and this difference was not significant (*p* = 0.4054). Comparison of SARI etiology did not reveal significant differences in influenza types and subtypes distribution between the males and the females in total: influenza A(H1N1)pdm09 was registered in 18.4% and 20.2%, influenza A(H3N2) in 17.5% and 15.3%, respectively. However, age-specific analysis of etiology shown that influenza A(H1N1)pdm09 cases were detected in males more often compared to females in age groups of 7–14 years, 15–17 years and > 65 years (27.1% against 18.6%, 21.6% against 8.1% and 15.9% against 6.8%, respectively). Such differences were not registered for influenza A(H3N2) infection. No significant differences in the percent of ORV between males and females in total (35.8% and 40.5%) were observed this season. In patients aged ≥ 65 years some causative agents (RSV, CoV) were detected this season only among women, whereas parainfluenza viruses and metapneumovirus were detected mostly among men.

### Co-morbidity in Patients with Influenza

**Fig. 3:**
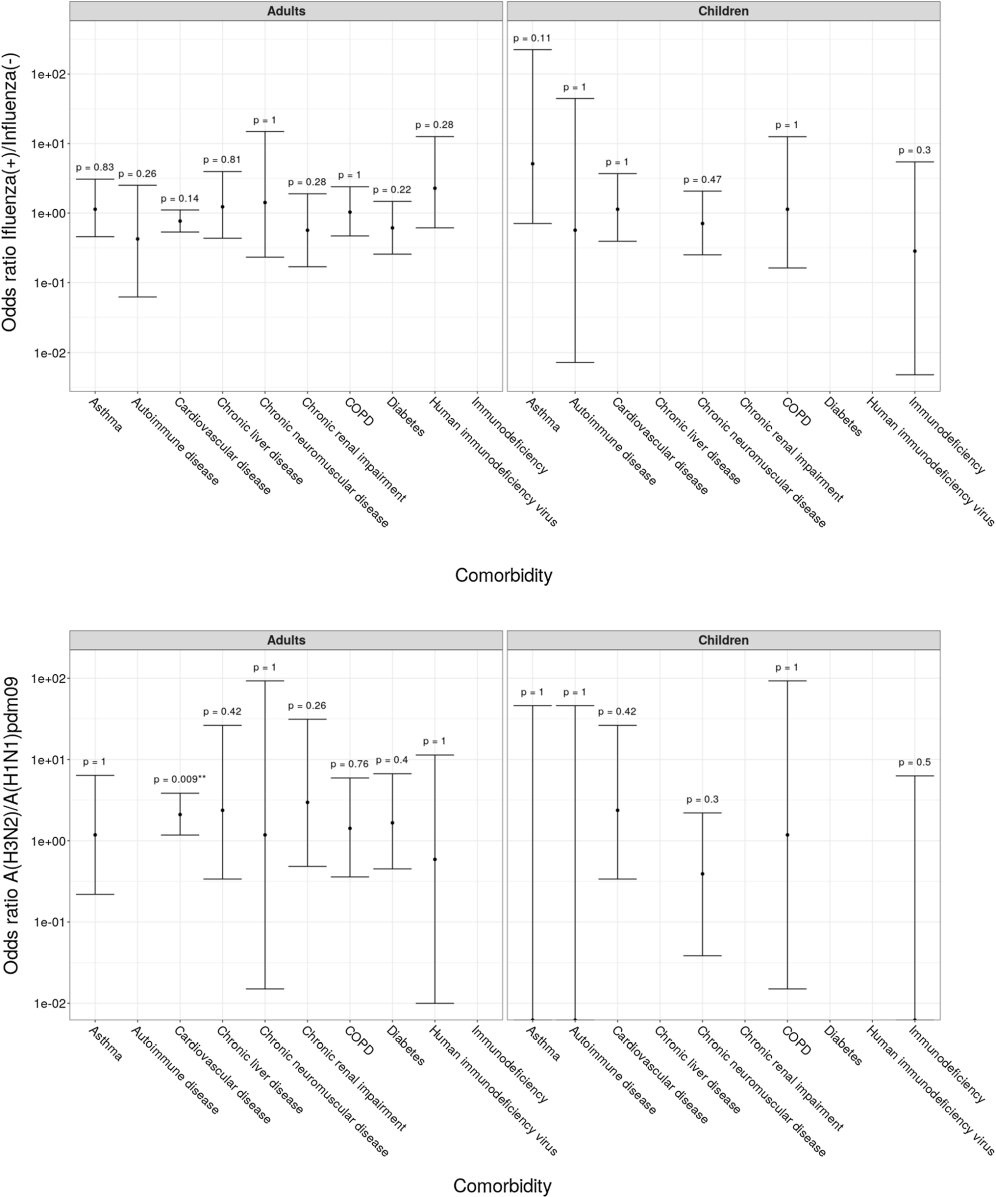
Odds ratios of comorbidities in patients hospitalized with influenza A(H1N1)pdm09 and A(H3N2) infections

Most of the hospital-admitted patients with laboratory-confirmed influenza (LCI) did not have any underlying chronic conditions. Only 138 (12.5%) of 1105 patients with LCI had comorbidities. Concomitant pathology was registered mostly among adult patients, with 117 (29.3%) cases. Only 21 (2.9%) cases were among children. The most common pathology was cardiovascular disease (CVD), which was registered in 63 patients with LCI (57 adults and 6 children). Those next in importance, in descending order, were chronic obstructive pulmonary disease (COPD, 12 LCI patients), diabetes (9 patients), asthma (9 patients), and chronic renal impairment (7 patients). In contrast, chronic neuromuscular diseases were found mostly in children with LCI. Comparative analysis of the rates of the indicated chronic comorbidities, among LCI and influenza-negative patients, showed that differences between these groups of patients were not statistically significant (*p* > 0.05). In patients with influenza A(H3N2) infection CVD and diabetes were 1.2–2 times more common than among influenza-negative patients, however, these differences were not significant. The only significant difference was found when comparing the OR in adult patients with CVD, which was higher in A(H3N2) influenza compared to influenza A(H1N1)pdm09 (Fig. 3).

### Influenza and ORV in Pregnant Women

A total of 129 pregnant women were included in the study. Influenza A viruses were detected in 80 (62%) pregnant. Percent detection of LCI in the control group was lower (46.7%). No significant differences were observed for influenza A(H1N1)pdm09 infection. However, influenza A(H3N2) was 1.5 times more common in pregnant women, compared to the adjusted by age not pregnant control group, these differences were also not significant (*p* > 0.05). No clear prevalence was observed in ORV detection among pregnant women, compared to the adjusted control group. Percent comorbidity, in pregnant women with LCI or other respiratory agents, did not differ significantly from the control group (Table 2).

**Table 2:** Influenza and other respiratory viruses detection among pregnant and non-pregnant women

Indices	Pregnant woman	Non-pregnant women (adjusted control)
Number of woman	129	75
Age group	17–42	15–42
Influenza virus detected (total)	80 (62.0%)	35 (46.7%)
A(H1N1)pdm09	32 (24.8%)	16 (21.3%)
A(H3N2)	48 (37.2%)	18 (24.0%)
A n/t	0	1 (1.3%)
ARI agent detected (total):	11 (8.5%)	9 (12.0%)
Parainfluenza virus	3 (2.3%)	0
Adenovirus	0	2 (2.7%)
RSV	0	1 (1.3%)
Metapneumovirus	3 (2.3%)	0
Coronavirus	2 (1.6%)	2 (2.7%)
Rhinovirus	3 (2.3%)	4 (5.3%)
Co-morbidity cases	17 (13.2%)	15 (20.0%)
Co-morbidity cases among flu positive women	10 (7.8%)	7 (9.3%)
Co-morbidity cases among other ARI agents	3 (2.3%)	3 (4.0%)

### Obesity

Obesity was found in 33 of 926 investigated cases and only among adult patients. Generally, LCI among patients with normal weight was registered approximately at the same frequency as in obese patients (43% vs. 45.5%). The obesity increased hospitalization rate with influenza A(H3N2) (33.3% vs. 24.7%), rhinovirus (9.1% vs. 4.0%), and metapneumovirus (6.1% vs. 1.3%) infection, however, these differences were not significant (*p* > 0.05).

### Severity Criteria Implementation

According to assessment of patient condition by doctors, the majority (92.7%) of patients hospitalized with symptoms of respiratory infection belonged to the ‘moderate severity’ category; ‘severe’ cases accounted for 7.2%, and ‘extremely severe’ cases accounted for only 0.13%. Analysis of the distribution of different severity criteria of respiratory disease, depending on causative agent, showed that hyperthermia (≥ 39 °C) was the most frequent clinical sign for influenza A(H1N1)pdm09 (*p* < 0.05). This symptom was significantly less common (*p* < 0.05) in patients with RSV, RhV, or MpV infection. In contrast, decreased blood O_2_ concentration, and dyspnea were more common with RSV, RhV, and MpV infection compared to influenza (*p* < 0.05). Hemorrhagic syndromes were more common with rhinovirus infection, and ICU admission was registered significantly more often with RSV infection, compared to influenza (*p* < 0.05) (Table 3).

**Table 3:** Implementation of severity criteria in evaluation of influenza and ORI among hospitalized patients

Criterion for disease severity	Registered # of patients	Influenza H1N1pdm *N* = 590		Influenza H3N2 *N* = 500		RSV *N* = 461		Rhino *N* = 251		MpV *N* = 163	
		*n*	%	*n*	%	*n*	%	*n*	%	*n*	%
Hyperthermia (> 39 °C)	1768	404	68.5	296	59.2	224	48.6	117	46.6	75	46.0
Hypoxia (SpO_2_ ≤ 94%)	161	24	4.1	24	4.8	23	5.0	15	6.0	26	16.0
Lethargy	11	1	0.2	1	0.2	0	0.0	0	0.0	0	0.0
O_2_ less than normal (95%–97%)	347	40	6.8	25	5	94	20.4	48	19.1	27	16.6
Dyspnea	659	104	17.6	72	14.4	188	40.8	73	29.1	72	44.2
Blood urea > normal^a^	79	7	1.2	15	3	6	1.3	2	0.8	2	1.2
Hemorrhagic syndrome	144	18	3.1	27	5.4	18	3.9	20	8.0	7	4.3
ICU admission	89	15	2.5	4	0.8	22	4.8	5	2.0	3	1.8
Mechanical ventilation	9	0	0.0	1	0.2	1	0.2	1	0.4	1	0.6

^a^Blood urea norm for children < 14 years is 1.8–6.4 mmol/l, for the patients aged ≥ 14 years is 2.5–6.4 mmol/l)

### Evaluation of Influenza Vaccine Effectiveness

A total of 95 (5.2%) patients, among 1834 patients aged ≥ 3 years, had been vaccinated, and 22 (23.1%) of them were positive for influenza. For the 2018–2019 epidemic season, influenza vaccine effectiveness (IVE) in prevention of patient hospitalization, evaluated using a test-negative case–control design, was determined to be 57.0% for children and 62.0% for adults. IVE in children increased from 42% in age group 3–6 years to 70.6% in group 7–17 years (Table 4).

**Table 4:** Influenza vaccine effectiveness against hospitalization by age groups

Age group (years)	Number of vaccinated patients	Vaccinated		Not vaccinated		Odds ratio OR	IVE %
		Flu (+)	Flu (−)	Flu (+)	Flu (−)		
3–6	31	9	22	225	318	0.58	42.0
7–17	29	5	24	127	179	0.29	70.6
Sub-total children	60	14	46	352	497	0.43	57.0
Sub-total adults	35	8	27	391	499	0.38	62.0
Total	95	22	73	743	996	0.4	60.0

**Fig. 4:**
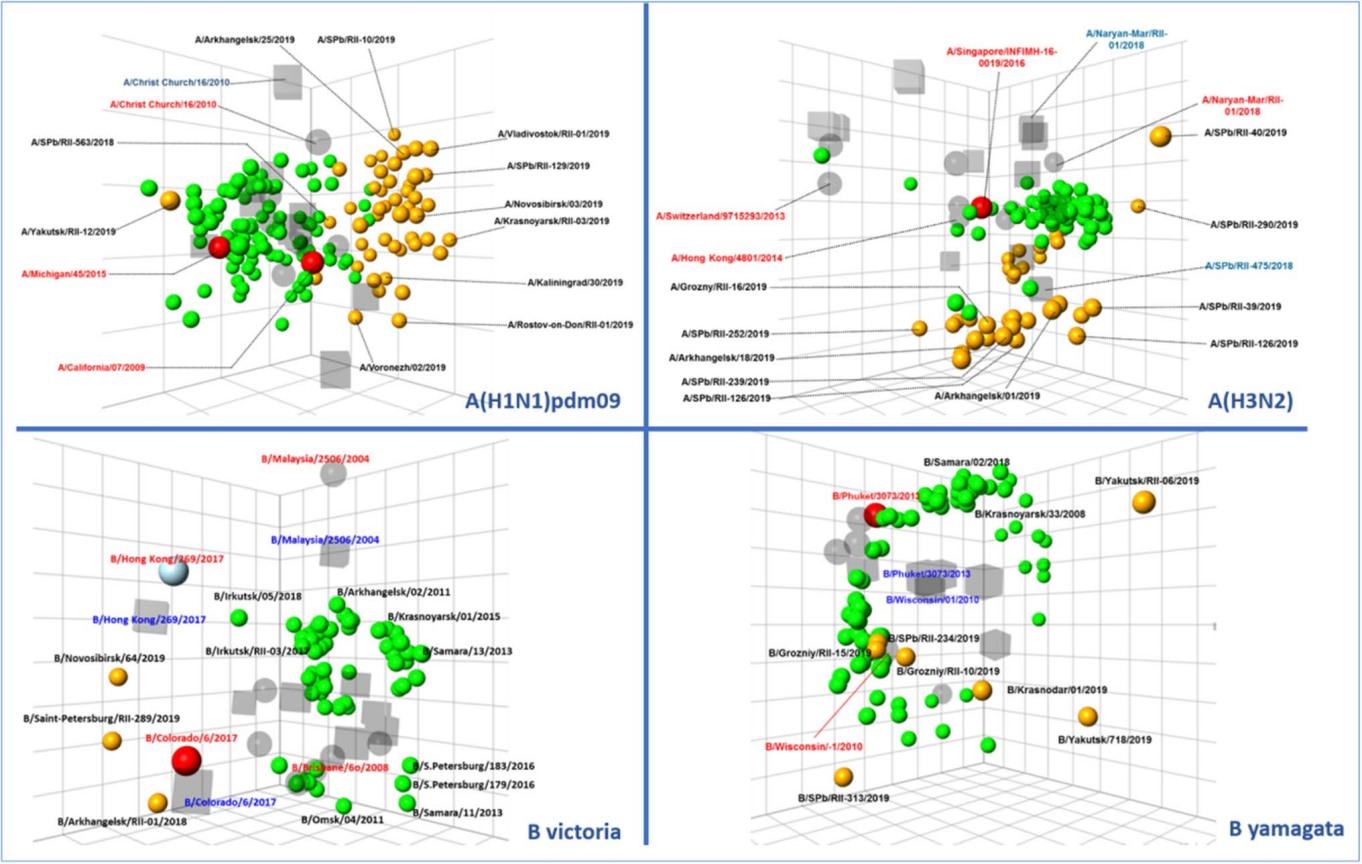
Three-dimensional antigenic map of influenza A(H1N1)pdm09 (on the left) and A(H3N2) (on the right) viruses that circulated in Russia in the season 2018–2019. NOTE: grey or red spheres indicated with red symbols—reference antigens; grey cubes/squares indicated with blue symbols—reference antisera; green spheres indicated with bold black symbols—old test antigens (2013–2017); orange circles—test antigens season 2018–2019. One map square represents a twofold difference in HI titre

Evaluation of IVE, by separate influenza A virus subtypes, showed that it was higher against influenza A(H1N1)pdm09, compared to influenza A(H3N2) (60.3% and 45.8%, respectively). This possibly could be due to delayed antigenic drift of influenza A(H1N1)pdm09 viruses and more heterogeneity of the influenza A(H3N2) population revealed by antigenic analysis of viruses circulating in Russia. All isolates A(H1N1)pdm09 analyzed in HI were antigenically closely related to the A/Michigan/45/2015 vaccine strain and belonged genetically to the clade 6B.1. The majority of influenza A(H3N2) viruses analyzed in HI or MN assay were well recognized by the ferret antisera raised against the A/Singapore/INFIMH-16-0019/2016 vaccine strain belonging to genetic group 3C.2a1b, however, about 7.5% of the viruses tested showed decreased reactivity with that antiserum. Antigenic mapping of influenza A(H1N1)pdm09 viruses isolated in Russia showed that strains of this season clustered around the reference vaccine strain A/Michigan/45/2015; the distribution of influenza A(H3N2) was more heterogeneous and the strains isolated from patients were dispersed more widely around the A/Singapore/INFIMH-16-0019/2016 vaccine virus (Fig. 4).

Phylogenetic analysis of influenza A(H1N1)pdm09 viruses showed that, during the 2018–2019 season, the percentage of viruses possessing the S183P substitution in HA1 increased. This mutation affects receptor-binding specificity (increasing binding to α2,6 SA-linked receptors). Most influenza A(H3N2) viruses belonged to clade 3C.2a1b. However, they were genetically heterogenous and split in three genetic subgroups with mutation in antigenic site A (T135N, T131K, or R142G) or in antigenic site E (E62G). The rare strains of Victoria lineage belonged to clade 1A, genetic subgroup Δ162-164 (B/Cote d'Ivoire/1662/2018-like viruses), and had additional G133R and K136E substitutions.

## Discussion

The expansion and standardization of clinical trials, the use of sensitive and specific molecular diagnostic methods (such as PCR detection of a whole variety of respiratory viruses causing SARI), and whole genome NGS analysis of influenza viruses are providing new information on the roles of viruses in the development of SARI and specific pathophysiological conditions. Ultimately, this will ensure the formation of new approaches to influenza vaccine improvement and to development of targeted antiviral therapies. In addition, PCR provides the ability to recognize age-specific etiology of severe respiratory infections (including those with comorbidity) and to evaluate possible risk factors. Our investigation covered the entire period of the influenza epidemic in Russia (from week 52.2018 to week 14.2019). The epidemic period featured co-circulation of influenza A(H1N1)pdm09 and A(H3N2), with minor contribution from influenza B/Yamagata and B/Victoria viruses. RSV was recognized as the most frequent causative agent of admission of young children which circulated simultaneously with influenza A viruses. Influenza A more often affected older children and adults. Conversely, ‘females aged 18–64 years’ were hospitalized more frequently than males. This may be due to hospitalization of pregnant women of childbearing age, among which influenza A(H3N2) was detected 1.5 times more often, compared to the adjusted control group. These results are consistent with data obtained in sentinel surveillance indicating that pregnancy is one of the leading risk factors for hospitalization with influenza [16]. The most significant SARI agents in ICU were RSV, influenza A(H1N1)pdm09 virus, and rhinovirus (28.1%, 9.2%, and 8.2%, respectively). The leading role of RSV was due to preferential ICU hospitalization of young children aged ≤ 2 years. Similar results regarding RSV predominance in young children have been reported in Norwegian children [17]. Globally, RSV was associated with 22% of all episodes of severe, acute lower respiratory infection, resulting in 55,000–199,000 deaths globally in children younger than 5 years old in 2005 [18]. In the United States, children aged < 1 year had the highest RSV hospitalization rate (2350/100,000) [19, 20]. As a result, WHO developed and published in 2019 WHO Strategy for Global RSV Surveillance Based on Influenza Platform. According this document the global burden of RSV-associated acute lower respiratory infections is estimated at 33 million annually, resulting in more than 3 million hospitalizations and 59,600 in-hospital deaths in children aged under 5 years. In infants under 6 months, RSV-associated acute lower respiratory infections account for about 1.4 million hospitalizations and 27,300 in-hospital deaths [21]. Many countries have recognized the importance of this pathogen and have established surveillance of RSV in certain settings. Russian Federation (Smorodintsev Research Institute of Influenza) and United Kingdom of Great Britain and Northern Ireland were included in the List of participating countries from the WHO European Region on the Pilot Phase. Monitoring of non-influenza respiratory viruses during the season showed that, in our investigation, RSV dominated during the entire period of increased influenza activity. Rhinovirus was the second most important agent in hospitalization (8.2% of all hospitalized patients and 5.6% of ICU-admitted patients). However, in previous years (2013–2014, 2016–2017), the rate of rhinovirus infection in ICU reached 16.7–18.9% (data not published). This agent is capable of exacerbating asthma in children [22, 23] and causing severe pneumonia in neonates [23]. It was shown earlier that adult patients with rhinovirus from RV-A or RV-B clusters were significantly more likely to have the composite outcome variable of 'death or ICU admission' [24], whereas cluster C was more pathogenic for children [26].

The most frequent criterion for severity in LCI patients was hyperthermia. In contrast, hypoxia, decreased blood O_2_ concentration, and dyspnea were more often reported with RSV and metapneumovirus infection. This can be explained taking into account the previously received radiographic findings, especially in high-resolution computer tomography, reflecting the histopathologic changes. It was shown, that RSV infection provokes plugging or occlusion of the bronchiolar airway lumens by sloughed necrotic and irregular epithelium and exudate, combined with peri‑bronchiolar infiltration, inflammatory cells and submucosal oedema. It was also established, that the biomarker CCL5, in the nasal epithelium during RSV bronchiolitis, is strongly predictive of physician-diagnosed asthma. Furthermore, single-nucleotide polymorphisms in genes coding for IL-8, IL-19, IL-20, IL-13, mannose-binding lectin, IFNG and RANTES, have been associated with wheezing following RSV infection in infants [27].

Influenza vaccine effectiveness (IVE) in prevention of patient hospitalization, for the 2018–2019 epidemic season, was evaluated in the GIHSN study using a test-negative, case–control design. The overall IVE values, for children and adults, were 57.0% and 62.0%, respectively. Overall IVE was higher against influenza A(H1N1)pdm09, compared to influenza A(H3N2) (60.3% and 45.8%, respectively). This correlates with delayed antigenic drift in the influenza A(H1N1)pdm09 virus: most influenza A(H1N1)pdm09 viruses were antigenically similar to the A/Michigan/45/15 vaccine strain and belonged to clade 6B.1 carrying additional a.a. substitutions in the Cb (S74R, I295V) and Sa (S164T) antigenic sites.

In contrast, high heterogeneity of the influenza A(H3N2) population was revealed by antigenic and genetic analysis. Most influenza A(H3N2) viruses were antigenically similar to the influenza A/Singapore/INFIMH-16-0019/2016 vaccine strain and genetically belonged to clade 3C.2a1b. However, they were divided into three genetic subgroups (3C.2a1b–T135K, 3C.2a1b–T131K, 3C.2a1b–T135N) based on a.a. substitutions in antigenic sites A and E.

Our study has shown significant IVE for both—children and adults—in preventing influenza cases that required hospitalization. As shown by long-term studies in U.S., Flu VE Network (2005–2018), and other studies, influenza vaccine effectiveness can vary widely (from 10 to 60%) based on study design, outcomes measured, population studied, and the season [26, 27]. According to our data on the 2018–2019 season, vaccination should be advocated to decrease the burden of influenza A(H1N1)pdm09-associated hospitalization and, to a lesser extent, the burden of influenza A(H3N2) admission. The results obtained are consistent with the data of other studies and indicate that monitoring for further evolution of circulating influenza viruses, including assessment of potential impact on vaccine protection, is warranted as postulated by WHO experts and many other scientists [30–32].

## Conclusions

1. RSV, influenza A(H1N1)pdm09 virus, and rhinovirus were the most significant causative SARI agents in hospitalization, especially among young children. These viruses circulated throughout the whole period of influenza activity and caused 28.1%, 9.2%, and 8.2% of ICU-admitted cases, respectively.
2. The most frequent criterion for severity in influenza patients was hyperthermia. In contrast, hypoxia, decreased blood O_2_ concentration, and dyspnea were more often reported with RSV and metapneumovirus infection.
3. For the 2018–2019 season, influenza vaccine effectiveness against pediatric and adult hospitalization were 57.0% and 62.0%, respectively. It was higher against influenza A(H1N1)pdm09, compared to influenza A(H3N2) (60.3% and 45.8%, respectively). According to antigenic and genetic analysis, these correlate with levels of matching viruses circulating in Russia and the strains included in vaccine composition.
4. These data indicated the necessity for new approaches to influenza vaccine improvement and the need to develop targeted antiviral therapies against influenza and RSV.

## Data Availability

The data that support the findings of this study are stored in an Electronic Database of Smorodintsev Research Institute of Influenza (responsible person K. A. Stolyarov) and available from the corresponding author, A. A. Sominina, upon reasonable request.

## References

[1] Global influenza strategy 2019–2030. Geneva: World Health Organization; 2019. Licence: CC BY-NC-SA 3.0 IGO, https://apps.who.int/iris/handle/10665/311184. Accessed 26 Oct 2021.

[2] Smeeth L, Thomas SL, Hall AJ, Hubbard R, Farrington P, Vallance P (2004). Risk of myocardial infarction and stroke after acute infection or vaccination. N Engl J Med.

[3] Wedzhicha JA, Seemungal TAR (2007). COPD exacerbations: defining their cause and prevention. Lancet.

[4] Baselga-Moreno V, Trushakova S, McNeil S, Sominina A, Díez-Domingo J, Draganescu A (2019). Influenza epidemiology and influenza vaccine effectiveness during the 2016–2017 season in the Global Influenza Hospital Surveillance Network. BMC Public Health.

[5] Puig-Barberà J, Mira-Iglesias A, Burtseva E, Cowling BJ, Serhat U, Sominina A (2019). Influenza epidemiology and influenza vaccine effectiveness during the 2015–2016 season: results from the Global Influenza Hospital Surveillance Network. BMC Infect Dis.

[6] Puig-Barbera J, Burtseva E, Yu H, Cowling BJ, Selim B, Jan K, Sominina A (2016). Influenza epidemiology and influenza vaccine effectiveness during the 2014–2015 season: annual report from the Global Influenza Hospital Surveillance Network. BMC Public Health.

[7] Simon B, Pichon M, Valette M, Burfin G, Richard M, Lina B (2019). Whole genome sequencing of A(H3N2) influenza viruses reveals variants associated with severity during the 2016–2017 season. Viruses.

[8] Domingo E, Sheldon J, Perales C (2012). Viral quasispecies evolution. Microbiol Mol Biol Rev.

[9] Dinis JM, Florek NW, Fatola OO, Moncla LH, Mutschler JP, Charlier OK (2016). Deep sequencing reveals potential antigenic variants at low frequencies in influenza A virus-infected humans. J Virol.

[10] Vasilijevic J, Zamarreño N, Oliveros JC, Rodriguez-Frandsen A, Gómez G, Rodriguez G (2017). Reduced accumulation of defective viral genomes contributes to severe outcome in influenza virus infected patients. PLoS Pathog.

[11] Puig-Barberà J, Tormos A, Trushakova S, Burtseva E, Sominina A, Pisareva M (2015). The global influenza hospital surveillance network (GIHSN): a new platform to describe the epidemiology of severe influenza. Influenza Other Respir Viruses.

[12] Commission of the European Union. Official Journal of the European Union 27.9.2012. Influenza virus—clinical criteria. 1.262/16 (2012).

[13] WHO manual for the laboratory diagnosis and virological surveillance of influenza. Geneva, WHO Press; 2011.

[14] Sominina AA, Burtseva EI, Lobova TG, Konovalova NI, Litvinova OM, Slepushkin AN, et al. Influenza virus isolation in cell cultures and identification: guidelines; approved by G. G. Onischenko, Head of the Federal Service on Surveillance for Customers’ Rights Protection and Human Well-Being, April 25, 2006.St.-Petersburg, 2006 (RU).

[15] Foppa IM, Haber M, Ferdinands JM, Shay DK (2013). The case test-negative design for studies of the effectiveness of influenza vaccine. Vaccine.

[16] Sominina AA, Smorodintseva EA, Stolyarov KA, Melnikova AA (2017). Enhancement of the influenza surveillance system in the Russian Federation: the main results of the sentinel surveillance for influenza and other acute respiratory viral infections. Epidemiol Vaccinal Prev.

[17] Moe N, Stensen IH, Krokstad S, Skanke LH, Risnes KR, Nordbo SA (2017). The burden of human metapneumovirus and respiratory syncytial virus infections in hospitalized Norwegian children. J Infect Dis.

[18] Shi T, McAllister DA, O'Brien KL, Simoes EAF, Madhi SA, Gessner BD (2017). Global, regional, and national disease burden estimates of acute lower respiratory infections due to respiratory syncytial virus in young children in 2015: a systematic review and modelling study. Lancet.

[19] Zhou H, Thompson WW, Viboud CG, Ringholz CM, Cheng PY, Steiner C (2012). Hospitalizations associated with influenza and respiratory syncytial virus in the United States, 1993–2008. Clin Infect Dis.

[20] Bawage SS, Tiwari PM, Pillai S, Dennis V, Singh SR (2013). Recent advances in diagnosis, prevention, and treatment of human respiratory syncytial virus. Adv Virol.

[21] WHO strategy for global RSV surveillance based on influenza platform (draft). Geneva: World Health Organization; 2019. pp. 1–49. https://www.who.int/publications/i/item/who-strategy-for-global-respiratory-syncytial-virus-surveillance-project-based-on-the-influenza-platform. Accessed 26 Oct 2021.

[22] Friedlander SL, Busse WW (2005). The role of rhinovirus in asthma exacerbations. J Allergy Clin Immunol.

[23] Bizzintino J, Lee WM, Laing IA, Vang F, Pappas T, Zhang G (2011). Association between human rhinovirus C and severity of acute asthma in children. Eur Respir J.

[24] Broberg E, Niemelä J, Lahti E, Hyypiä T, Ruuskanen O, Waris M (2011). Human rhinovirus C-associated severe pneumonia in a neonate. J Clin Virol.

[25] McCulloch DJ, Sears MH, Jacob JT, Lyon GM, Burd EM, Caliendo AM (2014). Severity of rhinovirus infection in hospitalized adults is unrelated to genotype. Am J Clin Pathol.

[26] Lau SK, Yip CC, Tsoi HW, Lee LY, So L, Lau Y (2007). Clinical features and complete genome characterization of a distinct human rhinovirus (HRV) genetic cluster, probably representing a previously undetected HRV species, HRV-C, associated with acute respiratory illness in children. J Clin Microbiol.

[27] Mammas IN, Drysdale SB, Rath B, Theodoridou M, Papaioannou G (2020). Update on current views and advances on RSV infection (Review). Int J Mol Med.

[28] Skowronski DM, Leir S, Sabaiduc S, Murti M, Dickinson JA, Olsha R (2019). Interim estimates of 2018/19 vaccine effectiveness against influenza A(H1N1)pdm09, Canada, January 2019. Euro Surveill.

[29] Centers for Disease Control and Prevention. CDC seasonal flu vaccine effectiveness studies. https://www.cdc.gov/flu/vaccines-work/effectiveness-studies.htm. Accessed 26 Oct 2021.

[30] World Health Organization (WHO). WHO recommendations on the composition of influenza virus vaccines. Geneva: WHO. https://www.who.int/influenza/vaccines/virus/recommendations/en/. Accessed 28 Oct 2020.

[31] Chambers C, Skowronski DM, Sabaiduc S, Winter AL, Dickinson JA, De Serres G (2016). Interim estimates of 2015/16 vaccine effectiveness against influenza A(H1N1)pdm09, Canada, February 2016. Euro Surveill.

[32] Skowronski DM, Chambers C, Sabaiduc S, De Serres G, Dickinson JA, Winter AL (2014). Interim estimates of 2013/14 vaccine effectiveness against influenza A(H1N1)pdm09 from Canada s sentinel surveillance network, 2014. Euro Surveill.

